# A Mouse Model for Binge-Level Methamphetamine Use

**DOI:** 10.3389/fnins.2016.00493

**Published:** 2016-11-02

**Authors:** Shkelzen Shabani, Sydney K. Houlton, Laura Hellmuth, Erika Mojica, John R. K. Mootz, Zhen Zhu, Cheryl Reed, Tamara J. Phillips

**Affiliations:** ^1^Department of Biology, Minot State UniversityMinot, ND, USA; ^2^Department of Behavioral Neuroscience and Methamphetamine Abuse Research Center, Oregon Health & Science UniversityPortland, OR, USA; ^3^VA Portland Health Care SystemPortland, OR, USA

**Keywords:** voluntary consumption, self-administration, selected line, genetic, withdrawal, abstinence, MALDR, MAHDR

## Abstract

Binge/crash cycles of methamphetamine (MA) use are frequently reported by individuals suffering from MA use disorders. A MA binge is self-reported as multiple daily doses that commonly accumulate to 800 mg/day (~10 mg/kg/day for a 170 pound human). A genetic animal model with a similar vulnerability to binge-level MA intake is missing. We used selectively bred MA high drinking (MAHDR) and low drinking (MALDR) mouse lines to determine whether several procedural variations would result in binge-level MA intake. Data were also collected in two progenitor populations of the MA drinking lines, the DBA/2J (D2) strain and the F2 cross of the D2 and C57BL/6J strains. The impact of 3 factors was examined: (1) concentration of MA in the two-bottle choice procedure used for selective breeding; (2) ratio of bottles containing MA vs. water, and (3) length of the withdrawal (or abstinence) period between MA drinking sessions. When MA concentration was progressively increased every 4 days in 20 mg/l amounts from 20 to 140 mg/l, maximum intake in MALDR mice was 1.1 mg/kg, whereas MAHDR mice consumed as much as 14.6 mg/kg. When these concentrations were tested in a multiple bottle choice procedure, the highest ratio of MA to water bottles (3:1) was associated with escalated MA intake of up to 29.1 mg/kg in MAHDR mice and 12.0 mg/kg in F2 mice; MALDR mice did not show a ratio-dependent escalation in MA intake. Finally, MAHDR and D2 mice were offered 3 bottles of MA vs. water at increasing concentrations from 20 to 80 mg/l, and tested under an intermittent 6-h withdrawal period, which was lengthened to 30 h (D2 mice) or to 30 or 78 h (MAHDR). D2 and MAHDR mice initially consumed similar amounts of 14–16 mg/kg MA, but D2 mice reduced their MA intake 3-fold after introduction of 30-h abstinence periods, whereas MAHDR mice retained their high level of intake regardless of withdrawal period. MAHDR mice provide a genetic model of binge-level MA intake appropriate for the study of associated MA-induced neurobiological changes and pharmaceutical treatments.

## Introduction

The path to problematic methamphetamine (MA) use leading to a diagnosable disease state is characterized in some individuals by gradual, persistent increases in use, often waxing and waning in so-called binge/crash cycles. Binge/crash cycles are well characterized for psychostimulants, and are thought to contribute to escalation of drug use, which is a hallmark of drug dependence (Roberts et al., 2013). A subgroup of MA users that were categorized as binge users, based on self-reported periods of high MA use until taking a break to sleep, also reported higher overall MA intake and more frequent use (Simon et al., 2002; Cheng et al., 2010). The likelihood of binge use is somewhat higher for younger MA users, though length of MA use seems not to have a significant effect on probability of binge use (Cheng et al., 2010). In other individuals, a more continuous pattern of use is typical, with MA use in the morning and at regular intervals throughout the day, rarely skipping days. This pattern is different from that of most cocaine users, which is more binge-like (Huber et al., 1997; Rawson et al., 2000; Simon et al., 2002). Amounts of MA consumed in a day on average for both types of users were between 0.3 and 0.8 g, though they could be as high as 1–4 grams in a binge cycle which lasts 1–3 days (Cho et al., 2001; Simon et al., 2002).

There is some evidence that genetic factors influence risk for a MA use disorder (e.g., Hong et al., 2003; Kinoshita et al., 2008; Uhl et al., 2008). However, lower rates of MA sampling, compared to some other drugs like nicotine and alcohol, impact the validity of genome-wide association studies in which a non-abusing control population is compared to a MA-dependent population. This is because many individuals in the control group are unlikely to have been exposed to MA, and thus, they may be mischaracterized. This is less of a problem for a drug like alcohol, which is widely sampled and readily available. Thus, other approaches to genetic investigation are important, including the use of genetic animal models in which drug history, environmental history, and genetic composition can be better controlled. Furthermore, despite extensive investigation, there are currently no food and drug administration-approved medications for the treatment of MA use disorder, and the identification of genetic risk and protective factors could lead to novel therapeutic targets (e.g., Harkness et al., 2015).

The gold standard for studying motivation to seek and self-administer MA in animals has been operant intravenous (IV) self-administration in rodents (Sanchis-Segura and Spanagel, 2006). However, studies exploring genetic risk for voluntary MA intake have been scant (de Wit and Phillips, 2012), in part, because rat or mouse models using IV procedures are not amenable to large scale genetic studies, which require large group sizes (e.g., ~120 animals per generation in a selective breeding project). MA administered through the oral route is readily absorbed in the digestive tract and, although details of the MA experience are different, is well documented to result in problematic levels of use similar to when administered through IV or nasal routes (Cruickshank and Dyer, 2009). Thus, we utilized a two-bottle choice MA drinking procedure to develop our mouse model of high and low genetic risk for MA use. Bidirectional short-term selective breeding methods (Belknap et al., 1997) were used to produce multiple replicate sets of MA drinking (MADR) selected lines in which one member of each set was MA high drinking (MAHDR) and the other was MA low drinking (MALDR). These mice have been used to identify genetic risk factors and genetically-correlated traits that are associated with protection from excessive MA intake (Phillips and Shabani, 2015).

The MAHDR lines of mice consume roughly 6 mg/kg/18 h on average from the 40 mg/l MA solution, whereas the MALDR lines consume roughly 0.4 mg/kg/18 h. In a 77 kg (or 170 lb) human, 500 mg/day would be in the middle of the common range of use, which is 6.5 mg/kg. Thus, in this relatively short duration MA intake model, relevant levels of intake are attained. Considerable data indicate that taste factors do not account for differences in MA intake between the MADR lines (Wheeler et al., 2009; Shabani et al., 2011), including data indicating that the lines consume comparable MA on the first day of access (Shabani et al., 2012a; Eastwood et al., 2014). In addition, only the MAHDR line establishes operant intracranial and oral self-administration of MA (Shabani et al., 2012a). However, for the purpose of characterizing neurochemical consequences and identifying therapeutic interventions, a longer term model with higher levels of intake is desirable.

In the current research, certain characteristics of the MA drinking procedure were manipulated to determine the impact on MA intake. The purpose was to identify a procedure(s) that would result in binge-like levels of consumption. Three factors were manipulated. First, the concentration of MA in the two-bottle choice procedure was progressively increased up to a concentration of 140 mg/l. Next, this manipulation was retained and the ratio of the number of bottles containing MA vs. water was varied across groups. The bottle ratio manipulation was based on data for ethanol drinking in which the amount of ethanol consumed by both C57BL/6J (B6; a high ethanol-consuming mouse strain) and 129X1/SvJ (a low to moderate ethanol-consuming strain) was positively related to the number of ethanol-containing bottles available (Tordoff and Bachmanov, 2003). This design has not been tried for MA. Finally, because human studies suggest that intermittency and cycles of abstinence have integral roles in the development of a drug use disorder, the length of the withdrawal period between MA access periods in our intermittent access procedure was manipulated. In general, in prolonged drinking procedures we predicted binge-like patterns of MA intake to emerge in the MAHDR mice. We specifically predicted that MAHDR mice would consume higher amounts of MA as MA concentration increased, in part, because lower volumes could be consumed to obtain a higher dose, but also because MAHDR mice exhibit low sensitivity to aversive effects of MA and high sensitivity to MA-conditioned reward and MA reinforcement (Wheeler et al., 2009; Shabani et al., 2011, 2012a,b; Harkness et al., 2015). We also predicted that more MA would be consumed by MAHDR mice when the ratio of MA to water bottles was higher, based on Tordoff and Bachmanov (2003). There are no free-choice MA intake studies that have examined the impact of withdrawal period. However, based on alcohol intake studies with DBA/2J (D2) and B6 mice (Cunningham et al., 2013; Dreumont and Cunningham, 2014), we predicted that short acute withdrawal periods within a day between MA access periods would sustain or even enhance MA intake, whereas longer withdrawal periods of days between MA access periods would attenuate MA intake.

## Materials and methods

### Animals

#### Methamphetamine drinking (MADR) lines

The MADR lines were generated from a reciprocal F2 cross of the B6 and D2 inbred strains, based on their voluntary MA intake in a two-bottle choice procedure. In this procedure, animals are given continuous access to a water bottle and access to 20 mg/l MA for 18 h/day for 4 days and then to 40 mg/l MA for another 4 days (Wheeler et al., 2009; Shabani et al., 2011). Based on average MA intake from the 40 mg/l solution, individuals with high and low intake were selected for breeding, with this procedure repeated across four generations of mice. Selection results for two replicate sets of lines, generated 2 years apart, have been described (Wheeler et al., 2009; Shabani et al., 2011). The MADR mice used for these studies were second or later litter offspring of fifth selection generation replicate 2 breeding pairs maintained within the Veterans Affairs Portland Health Care System (VAPORHCS) veterinary medical unit. All mice were MA naïve at the beginning of each study, and independent sets of mice were used in each experiment. Although significant sex differences have not been found in our previous studies of MA consumption, sex could have an impact when MA intake is greater, thus, equal numbers of male and female mice were used in each study. Mice for Experiments 1, 2, 3, and 5 were 108–118, 67–115, 63–117, and 74–97 days old, respectively.

#### DBA/2J and F2 cross mice

Male and female F2 cross mice (B6D2F2) were produced in the VAPORHCS veterinary medical unit. B6 and D2 mice were purchased from The Jackson Laboratory (Bar Harbor, Maine, USA) and reciprocal F1 crosses were paired to produce B6D2F1 and D2B6F1 offspring, which were then crossed to produce the F2 population tested in Experiment 2. Mice were 69–96 days old. For Experiment 4, male and female D2 mice were obtained from The Jackson Laboratory at 8 weeks of age, and were housed within the animal facility of Minot State University until testing began at 72 days of age.

### Housing and care

All mice were housed in shoe-box cages (31 × 20 × 15 cm; l × w × h) that were fitted with wire tops and lined with Bed-O-Cobs™ rodent bedding (The Andersons Inc., Maumee, OH, USA). Mice had free access to rodent chow (Purina 5001, 4.5% fat content; Animal Specialties Inc., Hubbard, OR or PicoLab® Laboratory Rodent Diet 5LOD, 4.5%fat content; Land O'Lakes Inc., St. Louis, MO, USA) and tap water. Room temperature was maintained between 20 and 22°C, and mice were housed on a 12:12 h light:dark schedule, with room lights turned on at 0600 h. All animals were acclimated to new housing environments for at least 2 weeks prior to an experiment. Animal care and use were approved by the Institutional Animal Care and Use Committees of the VAPORHCS or University of North Dakota and Minot State University and were conducted in accordance with the National Institutes of Health Guide for the Care and Use of Laboratory Animals.

### Drinking solutions

(+) Methamphetamine hydrochloride (MA) was obtained from Sigma (St. Louis, MO, USA), and used to make fresh drinking solutions every 4 days. MA drinking solutions were made by dissolving the appropriate amount of MA with the appropriate volume of tap water for the mg/l MA solutions utilized.

## Experiments

Experiments 1, 2, and 3 were conducted at the VAPORHCS, whereas Experiments 4 and 5 were conducted at Minot State University.

### Experiment 1. impact of increasing MA concentrations on MA intake in the MADR lines

A two-bottle choice procedure was conducted according to previous studies (Wheeler et al., 2009; Shabani et al., 2011), with the exception that MA concentration was increased beyond 40 mg/l in 20 mg/l increments up to 140 mg/l. Each concentration was offered vs. water for a 4-day period for 18 h per day. Forty-eight animals (12 per MADR line and sex) were weighed and individually housed on the first study day in plastic shoe-box cages with stainless steel wire tops (N10SS model; Ancare, Bellmore, NY, USA). Drinking bottles were 25-ml graduated cylinders fitted with stoppers and stainless steel sippers that were inserted between bars of the cagetops. Food was evenly distributed around the bottles, and one water bottle was provided at all times. In the first 48-h period of single housing, animals were acclimated to consuming fluid from the novel drinking bottles (one water bottle was offered at this time). Fluid consumption was determined every 24 h during this period, by measuring fluid levels in the graduated cylinders. Mice were then weighed and MA-containing bottles were placed on the cagetops during the 18-h period 3 h before the dark cycle began and 3 h into the light phase. Fluid levels were determined for both the 18-h period and the 6-h period, when only water was available. Body weight data were subsequently collected every 2 days. To avoid position bias, positions of the water and MA bottles were alternated every 2 days. Fluid consumption values and body weight data were used to determine mg/kg of MA consumed each day. Days 2 and 4 for each MA concentration were averaged to represent drinking for a particular MA concentration; this allowed the mice to identify the location of MA after a position switch and is consistent with the measure used during selective breeding.

### Experiment 2. impact of multiple-bottle choice on MA intake in the MADR lines

Procedures were identical to those for Experiment 1, with the exception that 48 MAHDR mice (6 per group and sex) and 24 MALDR mice (3 per group and sex) were offered 1–3 bottles of MA and 1–2 water bottles. Variation in the number of bottles across cages precluded alternating their positions, but MA concentration for all groups was increased every 4 days. Treatment groups are described in Table 1. Fewer MALDR mice were included in this study to reduce unnecessary animal usage, based on little impact of MA concentration on intake and low variability in Experiment 1. Likewise, fewer mice were tested per sex, based on the absence of a sex difference in Experiment 1 or in other MA drinking studies in these mouse lines (Wheeler et al., 2009; Shabani et al., 2011).

**Table 1:** **Groups for Experiments 2, 3, and 4**.

**Group**	**18-h bottle ratio MA:H_2_O**	**Number of bottles: 18-h period**	**Number of bottles: 6-h period (H_2_O)**
1	1: 1	2	1
2	1: 3	4	1
3	2: 2	4	1
4	3: 1	4	1

*H_2_O, tap water; MA, methamphetamine solution*.

### Experiment 3. impact of multiple-bottle choice on MA intake in B6D2F2 progenitor mice of the MADR lines

This experiment was conducted as a follow-up to Experiment 2 to compare results for the progenitor F2 mice, which consume an intermediate amount of MA to the MADR lines (Wheeler et al., 2009; Shabani et al., 2011). Procedures and groups (Table 1) were identical to those for Experiment 2. Seventy-two mice (9 per group and sex) were used in this experiment.

### Experiment 4. impact of withdrawal period on MA intake in D2 mice

**Figure 1:**
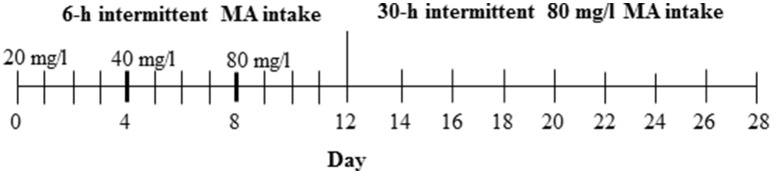
**Design for Experiment 4**. The control group had access to 4 water bottles (not shown), whereas the MA group had access to 3 MA bottles and 1 water bottle.

For this study, the D2 progenitor strain of the MADR lines, which consumes levels of MA approaching, but somewhat lower than, those of the MAHDR line (Eastwood and Phillips, 2012; Harkness et al., 2015), was tested to establish procedures prior to testing MAHDR mice, which were not as readily available. The 3 MA bottles and 1 water bottle group (Table 1) was chosen from Experiments 2 and 3, as the group with the greatest MA intake. Procedures for measurement of MA intake were as described for Experiments 1–3, except that D2 mice were weighed every 4 days (an unintentional change to the procedure; however, subsequent examination of body weight data collected every 2 days from other experiments indicated comparable data to every 4-day body weights), and the study was extended to address whether the length of MA withdrawal impacts subsequent MA consumption. Under 18-h per day access conditions, MA concentration was increased every four days from 20 to 40 and then 80 mg/l MA, followed by an additional 16 days, when the 80 mg/l concentration was offered every 30 h or every other day (Figure 1). The 30-h withdrawal period allowed for MA to be offered every other day and also retained the 18-h access period on the days on which MA was offered; thus, MA was withdrawn for 24 plus 6 h or 30 total h between MA access periods. Water was available at all times. Control group animals were offered the same number of fluid-filled bottles as the MA group, but all bottles were filled with tap water. Forty-eight mice (12 per group and sex) were used in this study.

### Experiment 5. impact of withdrawal period on MA intake in MAHDR mice

**Figure 2:**
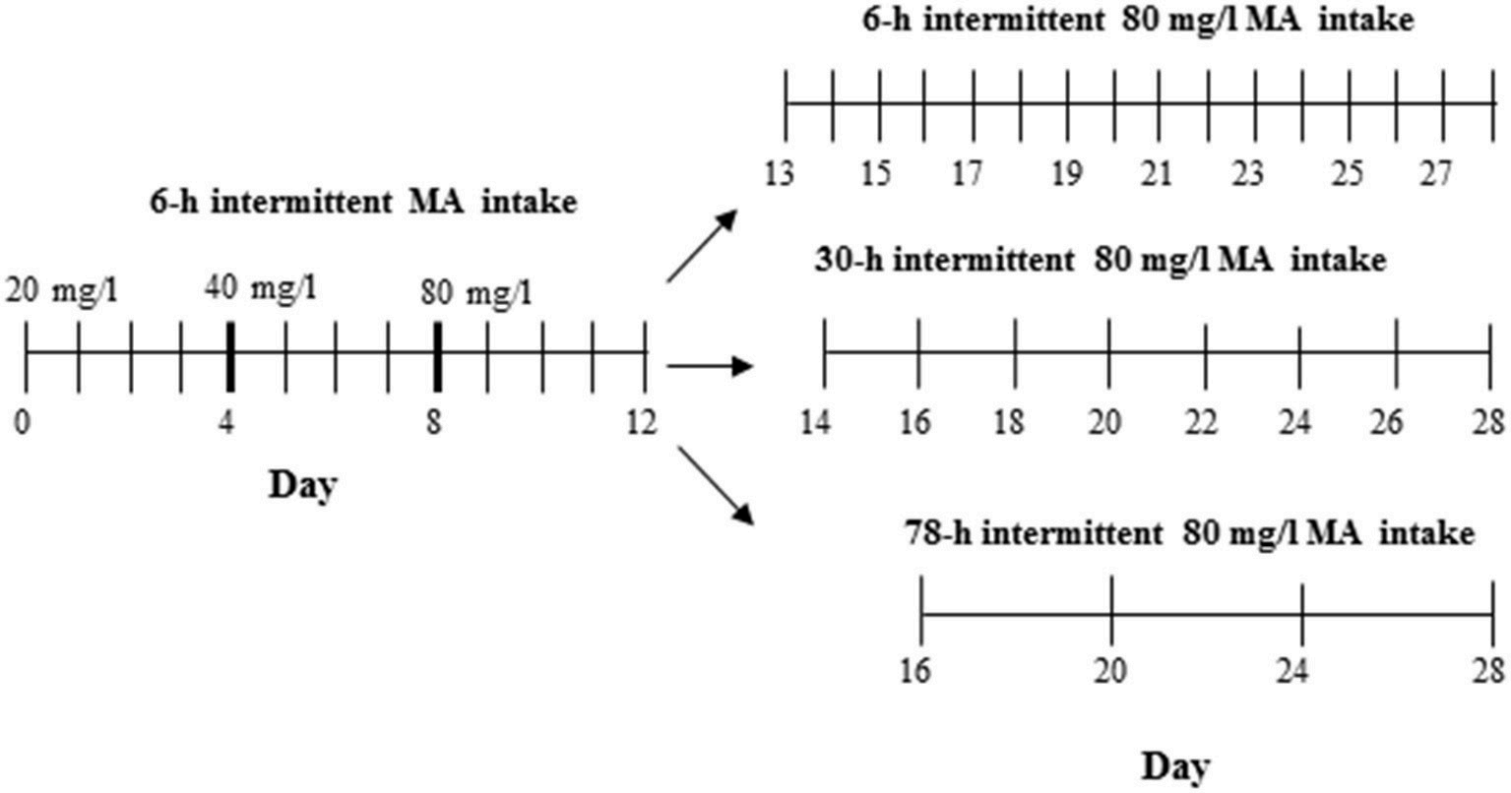
**Design for Experiment 5**. The control group had access to 4 water bottles (not shown), whereas the MA group had access to 3 MA bottles and 1 water bottle.

The procedure used to test MAHDR mice was the same as for Experiment 4 in D2 mice, with the exception that this experiment was extended to include multiple withdrawal durations after the first 4 days at 80 mg/l MA. Thus, one MA-drinking group continued with every day, 18-h access to 3 bottles of 80 mg/l MA so that the withdrawal period was the usual 6 h, whereas the other two groups were offered access either every other day as in Experiment 4 (30-h withdrawal period), or every 4 days (78-h withdrawal period). The procedure across days is illustrated in Figure 2. A control group was included that had access to only water. Fifty animals (6–7 per group and sex) were used.

### Data analysis

Data were analyzed using SPSS software (IBM® SPSS® Statistics software). The dependent variables were 18-h MA intake, and total 18-h volume intake. Total volume intake provided a measure of the effect of MA intake and of number of bottles on overall fluid consumption. Possible independent variables were MA concentration, line, group, and sex. Repeated measures ANOVA was used to determine the impact of increases in MA concentration and, in experiments 4 and 5, to also determine change in MA drinking pattern accross days. If the assumption of sphericity was not met, according to Mauchly's test (*p* <0.05), a conservative correction of the degrees of freedom, known as the Greenhouse-Geisser estimate, was used to assess signficance of the F-ratio. Significant 2-way interactions were followed up with analyses for simple main effects and then by *post-hoc* mean comparisons using the Tukey test, as appropriate. The level for significance (α) for all statistical tests was set at 0.05.

## Results

### Experiment 1. impact of increasing MA concentrations on MA intake in the MADR lines

**Figure 3:**
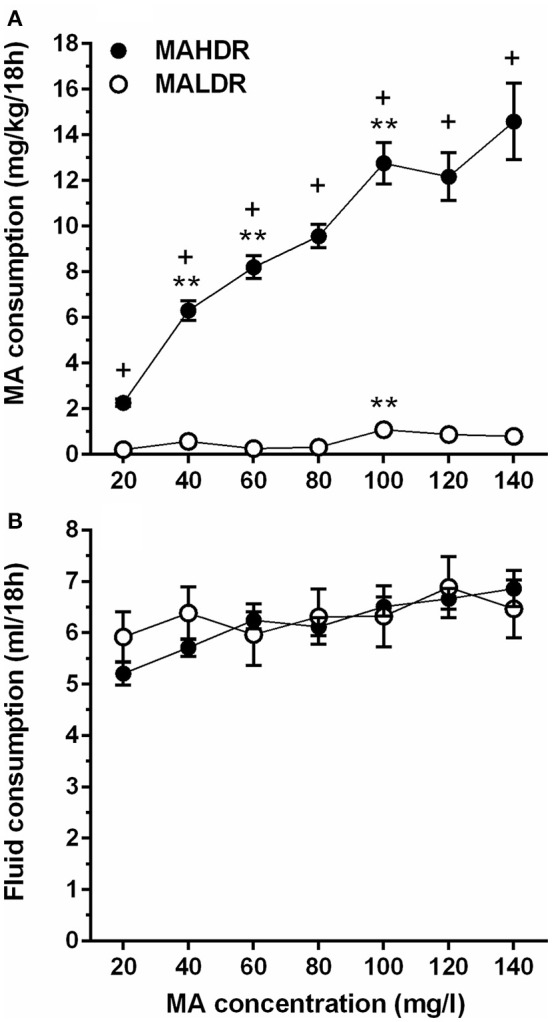
**Increasing concentration of MA leads to binge-level MA intake in selectively bred MA high drinking (MAHDR) mice. (A)** mean (± SEM) mg/kg/18 h MA consumed for MA concentrations offered for 4 days each and for each mouse line; B: mean (± SEM) total fluid consumed during the same 18-h periods for each MA concentration and mouse line. ^+^*p* <0.001 for the difference between MAHDR and MALDR at each concentration; ^**^p <0.01 compared to the next lower MA concentration.

In the initial line × sex × MA concentration repeated measures ANOVA, no significant effects involving sex were found; therefore, data were combined for the two sexes and analyzed for effects of line and MA concentration. Divergence in MA intake of the MADR lines increased with access to increasing concentrations of MA. MAHDR mice escalated their MA intake at each concentration, whereas the MA intake of MALDR mice was negligible at lower MA concentrations and increased only slightly at very high MA concentrations (Figure 3A). These characterizations were supported by the following statistical outcomes. There was a statistically significant line x MA concentration interaction [*F*_(2.4, 92.9)_ = 26.0, *p* <0.001], and simple main effects analysis revealed a significant effect of MA concentration within both the MAHDR line [*F*_(2.4, 48.0)_ = 29.4, *p* <0.001] and MALDR line [*F*_(3.4, 72.3)_ = 6.7, *p* <0.001]. Within-subjects contrasts of means between a lower MA concentration and a subsequent higher concentration for the MAHDR line revealed significant (all *p*s <0.01) increases in MA intake at 40, 60, and 100 mg/l MA concentrations. A similar analysis within the MALDR line revealed a significant (*p* <0.01) increase in MA intake only from the 80 to 100 mg/l concentration.

Total fluid consumption during the 18-h MA access period was also examined (Figure 3B). The MADR lines consumed similar combined amounts of fluid from the two bottles, although there was a significant main effect of MA concentration [*F*_(3.7, 151.5)_ = 7.8, *p* <0.001] that did not significantly interact with line. Follow-up within-subjects contrasts for data collapsed on line indicated a significant increase in fluid consumption (*p* <0.01) only when the MA concentration was increased from 20 to 40 mg/l.

### Experiment 2. impact of multiple-bottle choice on MA intake in the MADR lines

**Figure 4:**
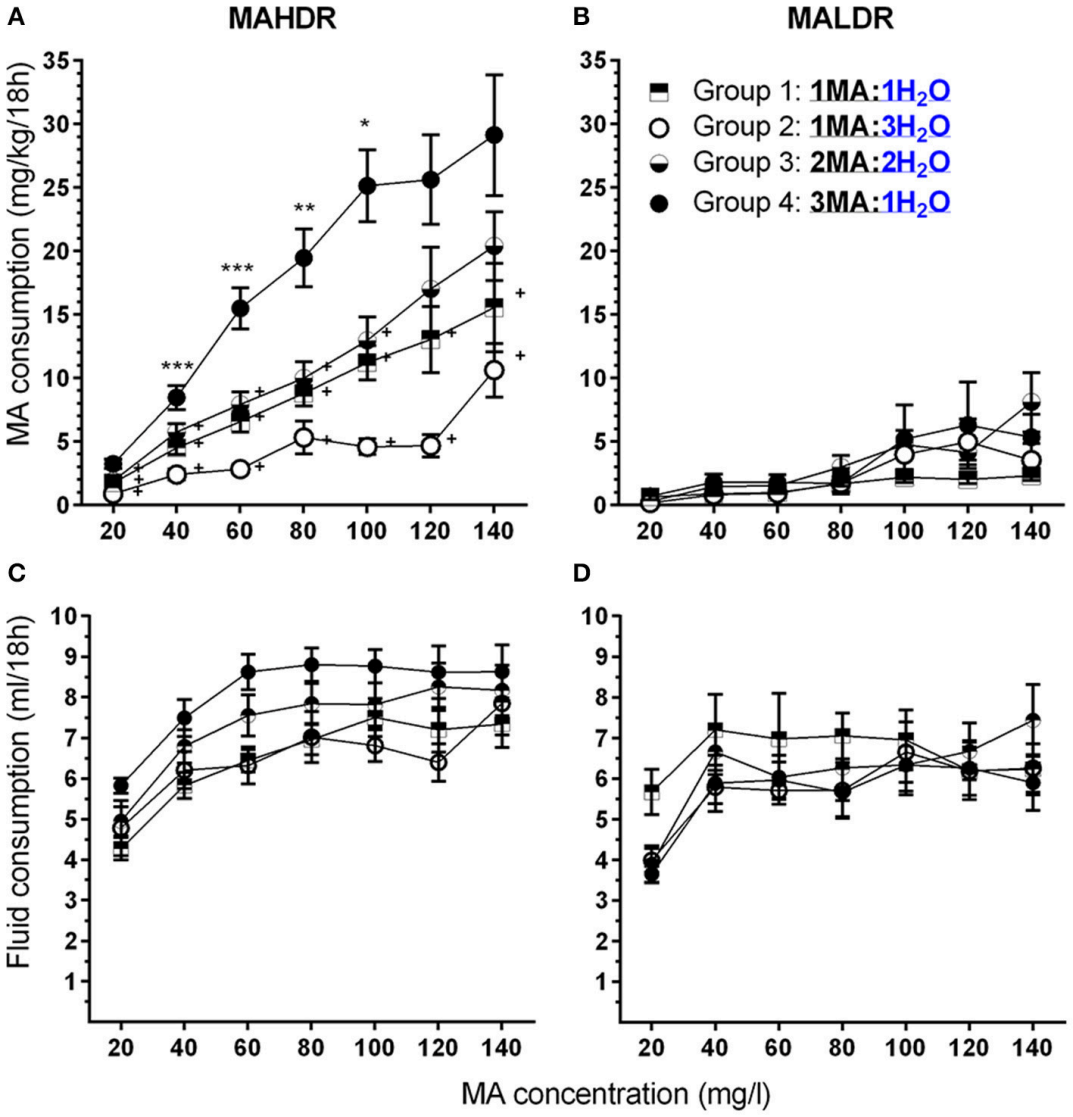
**A high MA bottle to water (H_**2**_O) bottle ratio (3MA:1H_**2**_O) increased MA intake in MAHDR, but not MALDR, mice. (A,B)** Mean (±SEM) mg/kg/18 h MA consumed for each MA concentration, mouse line, and group; **(C,D)**: Mean (±SEM) total fluid consumed during the same 18-h periods for each MA concentration, mouse line, and group. ^+^*p* <0.01 compared to Group 4 at each MA concentration; ^*^p <0.05, ^**^p <0.01, ^***^p <0.001 compared to the next lower MA concentration for Group 4 only.

Based on the absence of any significant effects of sex on MA intake, data were collapsed on this factor and analyzed by repeated measures ANOVA for the effects of line, group and MA concentration. As expected, MAHDR mice consumed significantly more MA than MALDR mice and the difference in amount consumed was dependent on MA concentration [Figures 4A,B; *F*_(2.3, 92.6)_ = 9.5, *p* <0.001 for the line × MA concentration interaction]. However, although the magnitude of the difference between the lines changed across concentrations, subsequent analysis with data collapsed on group indicated that MAHDR mice consumed more MA than MALDR mice at every concentration (all *p*s <0.001). In general, an increasing pattern of intake was seen across concentrations, but there was not a significant line × group × MA concentration interaction. However, for the purpose of model development it was important to determine if the various groups consumed significantly more MA across concentrations within-line and whether the MA to water bottle ratio had a significant impact on MA intake. Therefore, data were examined separately for each line.

Within the MAHDR line (Figure 4A), there was a significant group × MA concentration interaction [*F*_(6.0, 76.5)_ = 4.0, *p* <0.005]. Overall, Group 4 consumed more than twice as much MA across most of the MA concentrations, compared to any other group. For the effect of group within each MA concentration, significant group differences were detected at each concentration (*p*s <0.005), although MA intake significantly increased across concentrations in all groups (*p*s <0.001). To limit multiple mean comparisons, and because of our interest in developing a model of high MA intake, we considered group differences and concentration effects only with respect to Group 4, and indicate significant results in Figure 4A. Group 4 consumed more MA than all other groups for MA concentrations up to and including 100 mg/l and had significantly higher MA intake compared to Groups 1 and 2 for the 120 and 140 mg/l concentrations as well. Group 4 exhibited significant escalation of MA intake with each unit increase in MA concentration up to 100 mg/l MA, at which point MA intake plateaued at a mean (± SEM) level of 26.4 ± 2.2 mg/kg. Analysis of data for the MALDR line revealed only a main effect of concentration [*F*_(2.4, 35.5)_ = 10.1, *p* <0.001], but no significant main or interaction effect involving group (Figure 4B). Examination of the concentration effect with data collapsed on group indicated a significant increase in MA intake only from 80 to 100 mg/l, at which point MA intake plateaued at 4.45 ± 1.00 mg/kg (mean ± SEM).

Total fluid consumption (Figures 4C,D) was greater in MAHDR than MALDR mice [*F*_(1.0, 45.0)_ = 6.6, *p* <0.05 for the main effect of line] and there was a significant line × MA concentration interaction [*F*_(4.0, 179.0)_ = 4.5, *p* <0.005], but no significant effects involving sex or group. A significant effect of MA concentration was found for both lines (ps <0.01) and total fluid consumption of MAHDR mice increased significantly for MA concentration step increases up to 60 mg/l, and plateaued thereafter, whereas total fluid consumption for MALDR mice increased significantly only for the MA concentration increase to 40 mg/l.

### Experiment 3. impact of multiple-bottle choice on MA intake in B6D2F2 progenitor mice of the MADR lines

**Figure 5:**
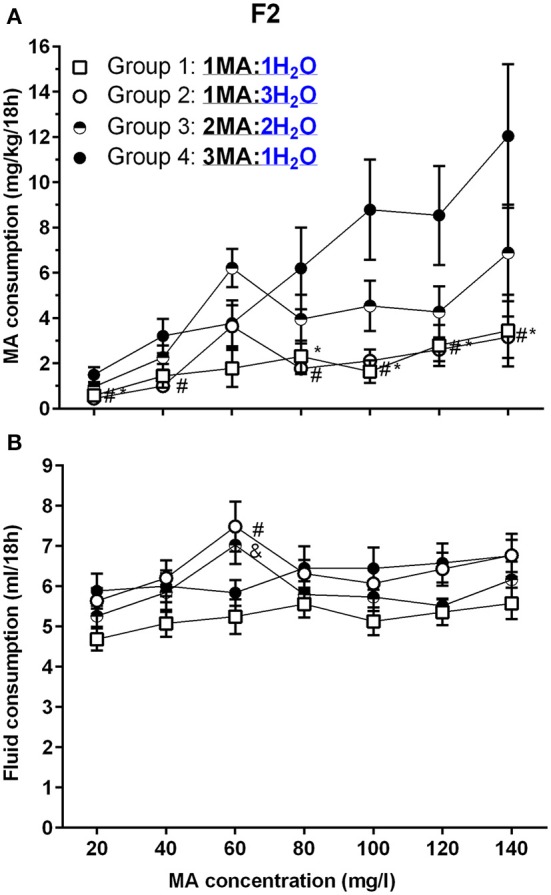
**A high MA bottle to H_**2**_O bottle ratio (3MA:1H_**2**_O) increased MA intake in B6D2F2 (F2) mice. (A)** Mean (±SEM) mg/kg/18 h MA consumed for each MA concentration and group; **(B)** Mean (±SEM) total fluid consumed during the same 18-h period for each MA concentration and group. ^*^, #, and & indicate *p* <0.05 for Group 4 compared to Group 1, 2, or 3, respectively.

There were no effects of sex in the initial analysis, so data for the sexes were combined and analyzed by repeated measures ANOVA for the effects of group and MA concentration. F2 mice increased their MA intake across concentrations (Figure 5A), but the increase was impacted by group [*F*_(4.7, 94.5)_ = 6.3, *p* <0.001 for the MA concentration × group interaction]. Significant group differences were detected at all concentrations (*p*s <0.05), and there were significant effects of concentration within each group (*p*s <0.05). Again, we performed follow-up mean comparisons within and relative to Group 4 only. Group 4 had higher mean MA intake at all MA concentrations, except 60 mg/l, compared to Groups 1 and 2, the groups that had access to a single MA bottle. MA intake for Group 4 did not differ from intake for Group 3, which had access to 2 MA bottles.

Total fluid consumption during the 18-h MA access period (Figure 5B) was not impacted by sex, but was affected by group and MA concentration [*F*_(11.6, 213.3)_ = 2.2, *p* = 0.01; for the MA concentration × group interaction]. There was a significant effect of MA concentration within each group (ps <0.05), and analysis of the effect of group for each MA concentration identified a significant group effect for total fluid intake only during the periods when 60 mg/l and 120 mg/l MA were offered (*p* <0.005 and *p* <0.05, respectively). Follow-up mean comparisons identified significant group differences only for 60 mg/l MA, as indicated in Figure 5B.

### Experiment 4. impact of withdrawal period on MA intake in D2 mice

**Figure 6:**
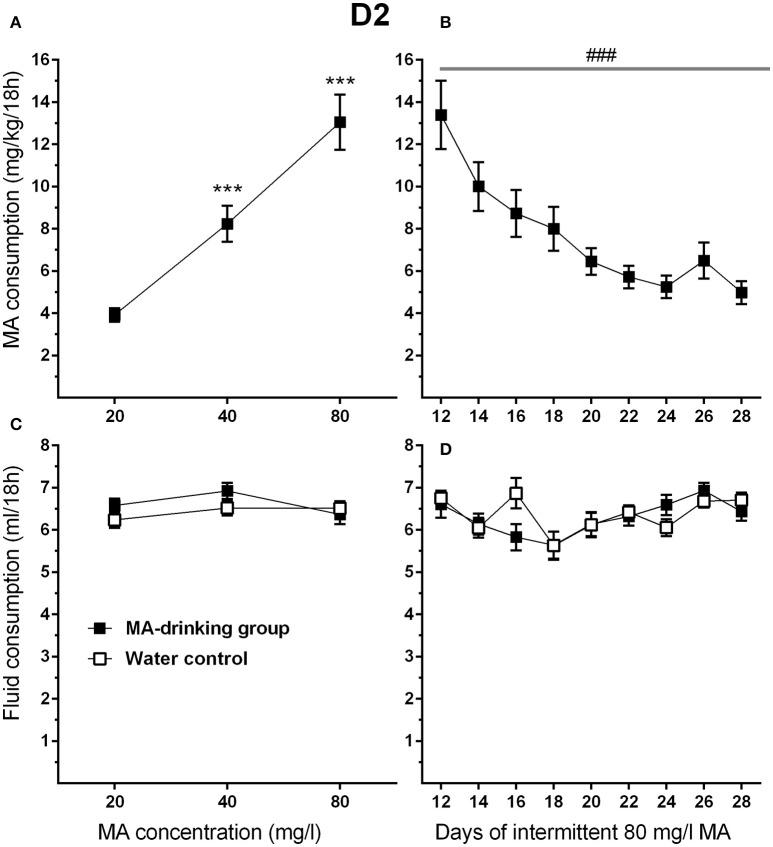
**MA intake is reduced in D2 mice when the intermittent MA withdrawal period is lengthened from 6 to 30 h. (A)** Mean (±SEM) mg/kg/18 h MA consumed during the MA drinking acquisition period; **(B)** Mean (±SEM) mg/kg/18 h MA consumed when access to 80 mg/l MA was separated by 30-h withdrawal (abstinence) periods; **(C,D)** Corresponding mean (±SEM) total fluid consumption for the MA and water only control groups. ^***^p <0.001, compared to the next lower MA concentration; ###*p* <0.001 for the main effect of Day.

To attain high levels of MA intake, only the four-bottle choice group that had access to 3 MA bottles and 1 water bottle, was used in this study. In addition, the highest concentration offered was 80 mg/l MA so that both increases and decreases in intake would be detectable during the increased withdrawal period phase of the study. MA drinking acquisition data were analyzed by repeated measures ANOVA for effects of sex and MA concentration. Similar to the results for MAHDR mice, D2 mice escalated their MA intake as MA concentration increased (Figure 6A). Significant main effects of MA concentration [*F*_(1.3, 28.9)_ = 56.4, *p* <0.001, and sex *F*_(1, 22)_ = 4.4, *p* <0.05] were found, but there was no significant interaction of these factors. Overall, females consumed more MA than males (mean ± SEM = 9.91 ± 1.02 and 6.89 ± 1.02 mg/kg/18 h for females and males, respectively, collapsed on concentration). During the MA drinking acquisition period, there were significant increases in MA intake from 20 to 40 mg/l MA (*p* <0.001) and 40 to 80 mg/l MA (*p* <0.001). Data from post-acquisition days 12–28 (Figure 6B) were analyzed separately to identify changes in MA intake associated with increased 30-h withdrawal period; day 12 was included as the final day on which animals had access to MA after the usual 6-h withdrawal period. D2 mice reduced their MA intake by more than half within the first 4 days following the 30-h withdrawal period (Days 14, 16, 18, and 20) and then their MA intake plateaued. A significant main effect of day [*F*_(2.5, 54.1)_ = 13.5, *p* <0.001], and a strong trend toward a sex effect [*F*_(1, 20)_ = 3.9, *p* = 0.07] were found, but there was no sex by day interaction. There were no statistically significant findings for total fluid consumption (Figures 6C,D).

### Experiment 5. impact of withdrawal period on MA intake in MAHDR mice

**Figure 7:**
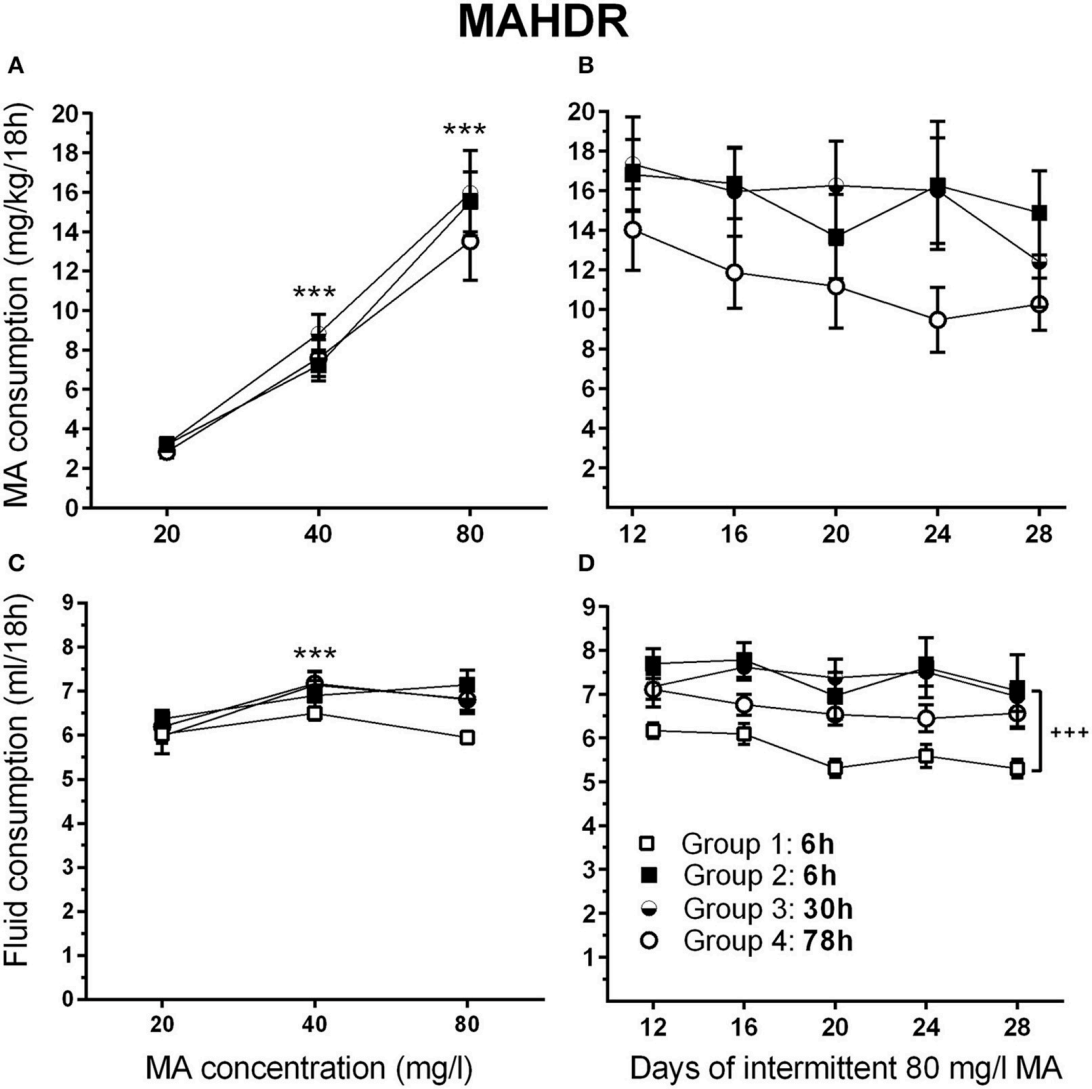
**MA intake remains high in MAHDR mice under multiple intermittent MA withdrawal periods. (A)** Mean (±SEM) mg/kg/18 h MA consumed for each MA concentration and group, during the MA drinking acquisition period; **(B)** Mean (±SEM) mg/kg/18 h MA consumed when access to 80 mg/l MA was separated by 6-, 30-, or 78-h withdrawal (abstinence) periods; **(C, D)** Corresponding mean (±SEM) total fluid consumed. Note: Group 1, 6 h (□) is water control group. The MA drinking group 2–4 designations are based on the variable withdrawal length after day 12. See Figure 1 for group information. ^***^p <0.001, compared to the next lower MA concentration; ^+++^*p* <0.001, Group 2–4 compared to Group 1.

There were no significant effects of sex in the initial analysis, so data for the sexes were combined and analyzed by repeated measures ANOVA for the effects of group and MA concentration. During the acquisition period, MAHDR mice escalated their MA intake, as indicated by a significant main effect of MA concentration [*F*_(1.2, 39.5)_ = 127.8, *p* <0.001]; however, the 4 intermittent access period groups (defined in Figure 2) were well matched for mean MA intake during this period, when they were on identical schedules (Figure 7A). Analysis of data for days 12–28, representing continued 6-h withdrawal or the transition from 6-h to 30-h or 78-h withdrawal, did not identify any significant group differences in MA intake (Figure 7B), but there was a main effect of day [*F*_(3.4, 120.2)_ = 3.7, *p* <0.01]. Mean comparisons indicated significantly lower MA intake on days 20, 24, and 28, compared to day 12.

Total fluid consumption during the acquisition phase changed significantly with concentration [*F*_(1.8, 83.8)_ = 17.9, *p* <0.001], but there were no effects of sex or group in the initial group × sex × MA concentration repeated measures ANOVA (Figure 7C). To examine the effect of concentration, data were collapsed on sex and group, and means for consecutively increasing MA concentrations were compared. There was a significant increase in fluid intake when MA concentration increased from 20 to 40 mg/l (*p* <0.001), but not from 40 to 80 mg/l (Figure 7C). Total fluid consumption during days 12–28 (Figure 7D) differed significantly across days [*F*_(3.6, 171.2)_ = 4.7, *p* <0.005] and groups [*F*_(3, 46)_ = 7.8, *p* <0.001], but there were no significant sex or any interaction effects found. Analysis of total fluid consumption for groups 2–4 revealed no differences between the groups and no drop in fluid intake across days. Mean fluid intake with the 3 MA access groups (Groups 2–4) collapsed revealed higher intake of 1.5 ± 0.09 ml (mean ± SEM) on average [*F*_(1, 48)_ = 19.2, *p* <0.001], compared to the water control (Group 1).

**Figure 8:**
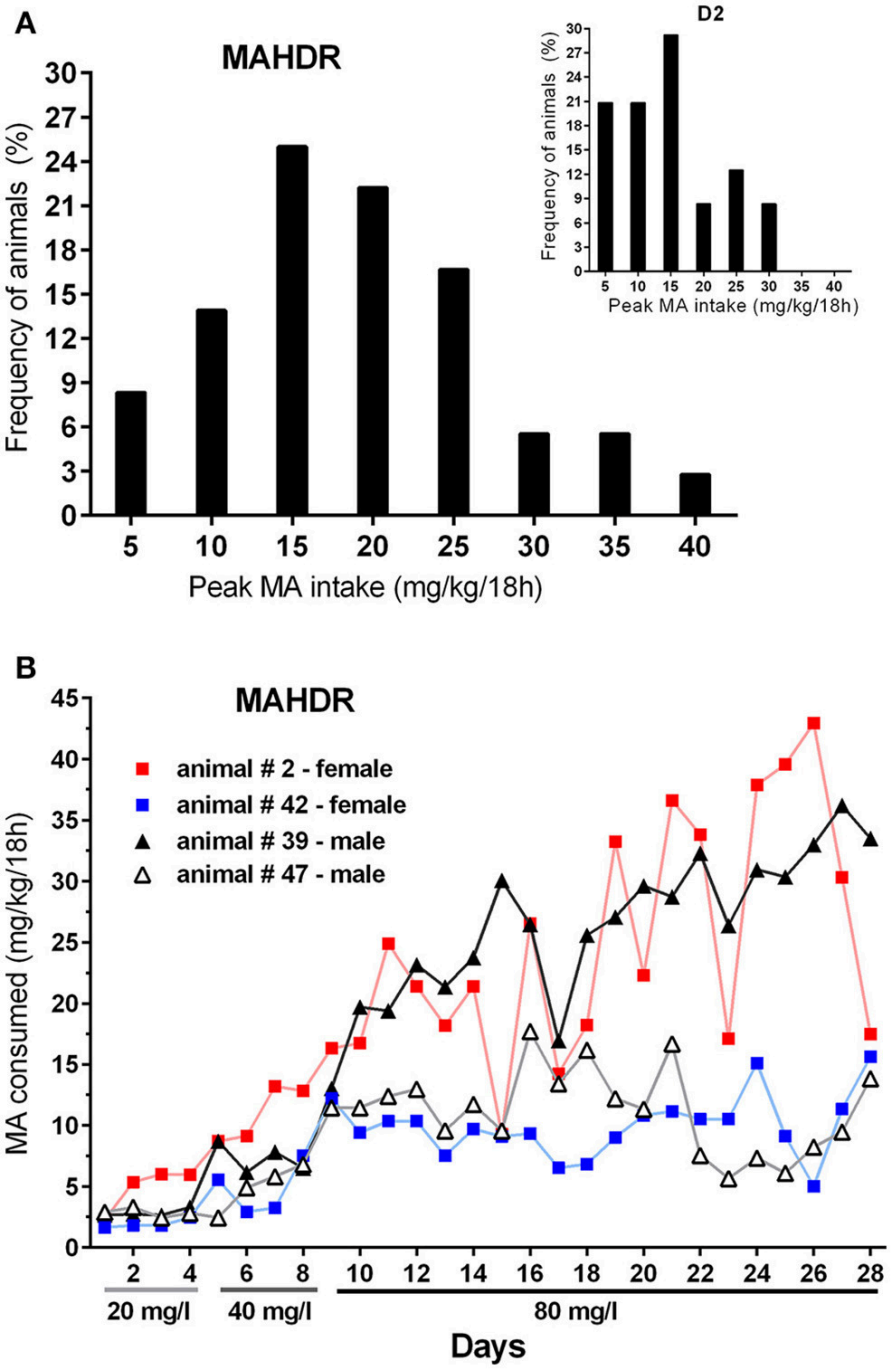
**MAHDR mice are variable in their peak MA intake and MA intake patterns across time. (A)** Frequency of animals in bins of 5 mg/kg/18 h, with peak 80 mg/l MA intake level ≥ the value along the x-axis. The inset shows the distribution for D2 mice from Experiment 4 for comparison; **(B)** MA drinking patterns of 4 MAHDR mice from Group 2 of Experiment 5 illustrating variability in patterns of peaks and troughs in binge-level MA intake. Patterns for Group 2 animals are shown, because they were given daily MA access, whereas the other groups were given MA access every other day or every 3 days.

Finally, to address pattern of MA intake at the individual level, data were examined for the frequency of MAHDR mice with amounts of peak MA intake from the 80 mg/l concentration arbitrarily set at above 15 mg/kg/18 h or below 10 mg/kg/18 h (Figure 8A). Eighty-five percent of the mice had peak intake greater than 15 mg/kg/18 h, and only 5% had peak MA intake below 10 mg/kg/18 h. The Figure 8A inset shows the same data for D2 mice from Experiment 4 for comparison; 58% of the D2 mice had peak intake greater than 15 mg/kg/18 h, and 21% had peak MA intake below 10 mg/kg/18 h, and the range of MA intake values is narrower in the D2 strain. The MA drinking patterns of 4 representative MAHDR mice (2 animals/sex) from Group 2 are represented in Figure 8B to illustrate the variability across animals. Group 2 is the 6-h withdrawal group that had access to MA every day, and represents the MA drinking procedure used during selective breeding for the 20 and 40 mg/l MA concentrations. Patterns are characterized by peaks of binge-level MA intake, in one animal of amounts sometimes greater than 40 mg/kg/18 h, followed by troughs, at times of orders of magnitude lower. Regardless of pattern, binge-like levels of intake can be seen across animals, with higher binge levels of MA intake associated with greater escalation as access is prolonged.

## Discussion

We have developed methods to study binge-level MA use patterns in a tractable genetic animal model. In this model, mice voluntarily consume amounts of MA that are similar to those reported in humans suffering from MA use disorders. To develop this model, we began with a mouse line that was selectively bred for higher levels of MA intake in a relatively short duration procedure that produced mean MA intake of ~6 mg/kg. By increasing the number of days of MA access along with progressively increasing MA concentration and increasing the ratio of MA to water drinking bottles to which the mice had access, remarkably high levels of MA intake were achieved. In a two-bottle choice procedure, MAHDR mice consumed almost 3 times more MA when the MA concentration was increased in a step-wise fashion to 120 mg/l, compared to their intake at 40 mg/l. Adding 2 more MA bottles to this procedure enhanced MA intake at every MA concentration in MAHDR mice. Although, MALDR mice exhibited a significant increase in MA intake (at only a single MA concentration), intake did not approach binge-like levels and was not dependent upon the number of MA bottles or the ratio of MA to water bottles. These procedural alterations also increased MA intake in the B6D2F2 progenitors of the MADR lines and in the higher MA-consuming D2 inbred strain. However, when a longer period of intermittent withdrawal was introduced to explore the potential impact of this manipulation on MA intake, the D2, but not MAHDR, mice precipitously reduced their MA intake. Hallmarks of drug disorders are escalation of use and maintenance of excessive use. Under certain test conditions, our existing MAHDR line models binge-level MA intake, and when contrasted with the MALDR line, provides a tool for identifying genetic and neuropharmacological mechanisms involved in susceptibility to and development of levels of MA use associated with MA use disorder.

The difference in MA intake between the MADR lines at all MA concentrations indicates that genetic factors responsible for divergence in MA intake during selection also contribute to the intake of higher MA concentrations. MA intake for the 20 and 40 mg/l MA concentrations in the current studies was virtually identical to that reported during the selective breeding of three independent replicate sets of MADR lines (Wheeler et al., 2009; Shabani et al., 2011; Harkness et al., 2015). Divergence in MA intake between the MADR lines increased as MA concentration increased, due to escalation of intake in the MAHDR line. However, it appears that a gradual increase in MA concentration may be important in this escalation of MA intake; MA intake of MAHDR mice in Experiment 5 for the 80 mg/l concentration was lower by ~5 mg/kg/18 h than in Experiment 2, possibly because the MA concentration was more precipitously increased in Experiment 5 (from 40 to 80 mg/l, skipping the 60 mg/l MA concentration). Whether such an abrupt change in MA concentration had aversive effects that reduced intake is not known. However, it is the case that MAHDR mice are particularly insensitive to the aversive effects of bolus MA treatments (Wheeler et al., 2009; Shabani et al., 2011, 2012b).

This is the first study to show that patterns of binge-level voluntary MA intake can be achieved using a simple multiple-bottle choice procedure in which the ratio of MA to water bottles is increased. Increasing access to MA with additional MA-containing bottles was based on the results of a 6-bottle choice ethanol study in which 2–5 ethanol bottles were available, in addition to one or more water bottles (Tordoff and Bachmanov, 2003). In that study, a substantial enhancement of ethanol intake was dependent upon the number of ethanol bottles. However, in the current MA studies, in mice that voluntarily consume MA, increasing the number of MA bottles was not enough to appreciably increase MA intake; rather the ratio of MA to water bottles was the important factor. Thus, animals that had a 1:1 ratio (i.e.; 1 MA:1 water or 2 MA:2 water) consumed similar amounts of MA, whereas a higher MA:water bottle ratio was associated with enhancement of MA intake, and a lower ratio with suppression of MA intake. That the MA to water bottle ratio did not significantly impact MA intake in the MALDR line highlights their general resistance to MA intake and indicates that genetic proclivity plays a role in the efficacy of this manipulation. Although the multiple bottle procedure had an impact on mean MA intake in the F2 mice, greater variability is clear in Figure 5A for this group of mice, which is comprised of individuals with different degrees of genetic risk for MA intake. In a two-bottle 4-h choice procedure in which MA drinking microstructure was investigated using a system that detects drinking spout contacts (lickometers), MAHDR mice had a greater number of MA drinking bouts, longer MA drinking bouts, shorter interbout intervals and shorter latency to the first MA drinking bout, compared to MALDR mice (Eastwood et al., 2014). Operant ethanol studies have linked shorter interbout interval and greater bout frequency with craving-like behavior (Samson et al., 1988), which we have yet to study in the MADR lines. In addition, we have yet to characterize blood MA level in our increasing MA concentration or multiple MA bottle procedures. In the microstructural analysis of MA intake, blood MA level at the end of a 4-h session corresponded with MA intake (Eastwood et al., 2014). Because mice are nocturnal and consume most of their food and fluid during the dark phase of the light:dark cycle, it is expected that most of their MA intake in the 18-h access procedure used in the current studies occurred during the dark phase. To determine the ideal time(s) to obtain blood samples that best reflect intake, we plan to use a lickometer system and obtain samples at multiple times in independent groups to avoid disrupting drinking behavior. By using independent groups, brain samples can also be obtained for determination of brain MA levels.

The progressive increase in MA intake by MAHDR mice to eventual sustained excessive intake has strong face validity for human binge-level use. Approximately 90% of MAHDR mice tested in our multiple bottle choice procedure consumed binge-like levels of MA daily. Humans exhibiting high levels of use take on average 800 mg/day, which for a 77 kg person translates to ~10 mg/kg/day; a majority of these individuals take MA at intervals of 2–4 h throughout the day (Rawson et al., 2000; Simon et al., 2002). This pattern of intake is remarkably different from that for cocaine, which is characterized by a more discontinuous or intermittent pattern, often with higher doses taken in the evening and on fewer days per month (Simon et al., 2002; Rawson et al., 2000). Approximately 40% of MA users in one study engaged in binge-taking cycles characterized by multiple episodes of daily use until use was terminated due to lack of sleep or lack of access to MA (Cheng et al., 2010). According to estimates, heavy users can take as much as 2–4 grams of MA in a binge cycle that lasts 1–3 days (Cho and Melega, 2002). Human deaths associated with toxic MA blood levels have been reported to be particularly prevalent among individuals that use MA via the oral route (Inoue et al., 2006). Accumulation of toxic levels of MA in the blood when orally administered is more likely because of the relatively slow absorption in the digestive tract and also slower onset of pharmacological effects (Schep et al., 2010), coupled to the generally slow clearance of MA from the blood in humans (Cruickshank and Dyer, 2009). According to Inoue et al. (2006), MA poisoned patients were very often diagnosed with edema in multiple organs. In our multiple bottle choice procedure, some MAHDR mice had extreme peaks of MA intake of up to 40 mg/kg/18 h. Importantly, patterns of MA intake in MAHDR mice were indicative of binge/crash-like intake, in which peaks lasted for 2–5 days followed by troughs of considerably lower amounts. However, there were no animal deaths and there was no significant difference in body weight between any of the groups including control mice (those drinking water only). Rodents metabolize MA more rapidly than do humans (Riviére et al., 2000; Cho et al., 2001), so amounts may not accumulate to as high a level. However, chronic kidney disease and hypertension are frequently found (>90%) among MA users (Jones and Rayner, 2015), and it will be important to assess blood and brain MA levels, MA clearance, and physical and physiological consequences of MA intake at the levels achieved in our animal model. Whether tolerance in neurochemical pathways that motivate MA intake plays a role in increasing MA consumption is not currently known.

The level of MA intake achieved using our drinking procedure is comparable to or greater than that obtained in rats using longer-access operant IV self-administration procedures (Jang et al., 2013). Rats with 6-h access to MA via the IV route, escalated and maintained high MA intake levels, whereas rats with 1-h access did not. Similarly, rats with longer access than 6 h in an operant IV procedure escalated and sustained high levels of MA self-administration (Cornett and Goeders, 2013). Rats were placed in operant boxes for 96 h/session for a total of 5 sessions separated each time by 72 h. While in the operant boxes, the animals were restricted to certain amounts of food intake and allowed to voluntarily self-administer 0.06 mg/kg infusions of MA under a fixed-ratio 1 schedule of reinforcement. Restriction of food intake may have enhanced drug intake in this study (Carroll et al., 1979; Carroll and Stotz, 1983). The rats self-administered ~75 mg/kg/96 h in week five, or a dose of about 0.8 mg/kg/h, which is similar to the 1.25 mg/kg/h obtained in the Jang et al. (2013) study. This MA intake level in the 96-h access study translates to ~14 mg/kg/18 h, which is relatively high MA intake, though somewhat lower than found in our 4-bottle choice genetic model. The authors stated that there was no MA intake escalation or change in binge/crash-like behaviors when the procedure was continued for longer than 5 weeks (Cornett and Goeders, 2013).

Our results indicate that there are individual differences in MA consumption pattern within our genetically selected MAHDR mouse line. Such differences could arise from genetic, environmental or both influences. MA consumption patterns in humans with MA use disorders are also highly variable, such that some individuals report frequent binge/crash cycles of use and consume a gram or more in a binge, whereas others report continuous MA use at lower levels of around 0.3 grams per day (Simon et al., 2002). In the current study, the initial MA intake of individual MAHDR mice at lower MA concentrations was more homogeneous, but MA intake diverged by orders of magnitude over time at a higher MA concentration (e.g., Figure 8B). Also, while MAHDR and D2 mice consumed similar amounts of MA when the intermittent abstinence period was 6 h in a 2- or 4-bottle choice procedure, only the MAHDR mice sustained their high levels of MA intake, when the intermittent abstinence period was lengthened to 30 h. Acute withdrawal effects within a couple of days of drug abstinence are thought to contribute to compulsive drug use (Koob and Le Moal, 2005; Jang et al., 2013; Whitfield et al., 2015). The peak of vulnerability to relapse of MA use in humans is after 24 h and drops linearly, but stays high for up to 10 days (McGregor et al., 2005; Zorick et al., 2010). However, MA metabolism is slower in humans than rodents, which could impact the duration of vulnerability. Humans that report binge use of MA have a higher frequency of MA use and higher MA intake (Cheng et al., 2010), though data appear to be lacking that indicate how stable MA use is from binge to binge. MAHDR mice with high binge-level peaks of MA intake when MA was available at a higher concentration, appeared to escalate their MA intake, as compared to MAHDR mice with more moderate peaks of MA intake (Figure 8B). This suggests that binge-level intake contributes to progressively higher MA intake in this model.

The difference in MA intake between the MAHDR and D2 mice when a longer period of MA withdrawal was introduced may be due to differences in sensitivity to acute withdrawal effects over time. MA withdrawal is characterized by fatigue, anhedonia, depression, and enhanced craving for MA (Rawson, 2013; Ren et al., 2015). In humans, regardless of the route of administration, MA has a long plasma half-life of 9–12 h, whereas subjective effects diminish after 4 h (Cho et al., 2001; Cruickshank and Dyer, 2009). In rodents, MA has a much shorter half-life of 70 min, whereas locomotor effects last for 3 h or less (Riviére et al., 1999, 2000; Cho et al., 2001; Shabani et al., 2012b). DBA/2Ha (Harlan, Dublin, VA) mice exhibited robust depression-like symptoms, as measured by the tail suspension test, after a 24-h acute withdrawal from a 7-day regimen of 5 or 10 mg/kg/day amphetamine infusion via minipump (Cryan et al., 2003). To our knowledge, sensitivity to the effects of acute MA withdrawal is not known in our D2 mice (from The Jackson Laboratory) or in MAHDR mice, but could differ and impact MA intake patterns. The level of MA intake of D2 mice was maintained when the intermittent abstinence period was 6 h, but dropped linearly when the abstinence period was 30 h. Likewise, for operant intragastric ethanol infusion, when compared to controls, D2 mice had higher ethanol intake within the first 24 h of acute withdrawal, but when the abstinence period was longer than 24 h, D2 mice precipitously reduced their subsequent ethanol intake (Cunningham et al., 2013). MAHDR mice sustained a high level of MA intake regardless of withdrawal period. Thus, genetic differences between D2 and MAHDR mice resulting from selective breeding may contribute to sustained excessive MA intake. In the current study, total fluid consumption between the water control and MA groups did not differ during the MA drinking acquisition period in either MAHDR or D2 mice, but MA group MAHDR consumed more total fluid than control group mice during the abstinence period manipulation phase. It is possible that the MAHDR mice experienced greater behavioral activation than D2 mice, corresponding with their greater MA intake, and perhaps greater thirst. Locomotor activation after 1-h operant oral MA self-administration sessions has been reported in the MAHDR line that corresponded with amount of MA consumed (Shabani et al., 2012a), but has yet to be examined under the current conditions.

In summary, our genetic mouse model of binge-level MA intake has some unique advantages. The mice are genetically vulnerable to excessive MA intake, and escalate and maintain their intake at binge-like levels similar to those reported in humans. There are differences among susceptible individual mice in pattern of intake and escalation, as would be expected among susceptible humans. The oral intake model is amenable to studies requiring larger numbers of animals (e.g., pharmacological studies with multiple drug doses) and for longer duration studies, because it avoids catheter implantation surgeries and complications associated with the IV route of self-administration, although slower absorption via the oral route (Schep et al., 2010) impacts the rate at which brain responses (e.g., dopamine increases) may occur. No food restriction, training or sweetener is necessary with our mouse model, as is often required with operant IV or oral studies. Animals can voluntarily control their intake at the microstructural level, which is similar to how humans control their MA intake, but taste factors need to be taken into consideration; no differences in the consumption of bitter, sweet or salty substances have been found in the MADR lines (Wheeler et al., 2009; Shabani et al., 2011, 2012b). Future studies will consider individual differences in the emergence of binge/abstinence cycles that are thought to increase drug-seeking behaviors associated with drug use disorders (for review see, Roberts et al., 2013), with a particular focus on genetic influences and behavioral, neural and physiological consequences.

## Author contributions

SS: experimental design, acquisition of some of the data, analysis and interpretation of all the data, and wrote the manuscript interactively with TP. SH, LH, and EM acquisition and some analysis of data, gave input to interpretation of data in experiments 4 and 5, and gave input to the manuscript pertaining to the same experiments. JM and ZZ: acquisition and some analysis of data, involved in conversations regarding interpretation of experiments 1–3. CR: development of experimental protocol and supervision of technical support, some statistical analysis of experiments 1–3, and manuscript editing. TP: experimental design, analysis and interpretation of all the data, and wrote the manuscript interactively with SS.

### Conflict of interest statement

The authors declare that the research was conducted in the absence of any commercial or financial relationships that could be construed as a potential conflict of interest.
